# Acetylcholine decreases formation of myofibroblasts and excessive extracellular matrix production in an in vitro human corneal fibrosis model

**DOI:** 10.1111/jcmm.15168

**Published:** 2020-03-16

**Authors:** Marta Słoniecka, Patrik Danielson

**Affiliations:** ^1^ Department of Integrative Medical Biology Umeå University Umeå Sweden; ^2^ Department of Clinical Sciences, Ophthalmology Umeå University Umeå Sweden

**Keywords:** collagens, cornea, fibrotic markers, keratocytes, scarring

## Abstract

Acetylcholine (ACh) has been reported to play various physiological roles, including wound healing in the cornea. Here, we study the role of ACh in the transition of corneal fibroblasts into myofibroblasts, and in consequence its role in the onset of fibrosis, in an in vitro human corneal fibrosis model. Primary human keratocytes were obtained from healthy corneas. Vitamin C (VitC) and transforming growth factor‐β1 (TGF‐β1) were used to induce fibrosis in corneal fibroblasts. qRT‐PCR and ELISA analyses showed that gene expression and production of collagen I, collagen III, collagen V, lumican, fibronectin (FN) and alpha‐smooth muscle actin (α‐SMA) were reduced by ACh in quiescent keratocytes. ACh treatment furthermore decreased gene expression and production of collagen I, collagen III, collagen V, lumican, FN and α‐SMA during the transition of corneal fibroblasts into myofibroblasts, after induction of fibrotic process. ACh inhibited corneal fibroblasts from developing contractile activity during the process of fibrosis, as assessed with collagen gel contraction assay. Moreover, the effect of ACh was dependent on activation of muscarinic ACh receptors. These results show that ACh has an anti‐fibrotic effect in an in vitro human corneal fibrosis model, as it negatively affects the transition of corneal fibroblasts into myofibroblasts. Therefore, ACh might play a role in the onset of fibrosis in the corneal stroma.

## 1 INTRODUCTION

Corneal scarring arises due to overproduction, excessive deposition and contraction of extracellular matrix (ECM).^1^ A regenerative wound healing process in the cornea of the eye, post‐surgery or after injury or infection, will result in restoration of normal structure and function of the cornea.^2^ However, scarring might occur in some cases, leading to fibrosis and corneal blindness.^3^

Transforming growth factor‐β1 (TGF‐β1) is a cytokine essential for the induction of the fibrotic response. In an uninjured cornea, TGF‐β1 is stored inside the corneal epithelium and is responsible for maintaining corneal integrity and wound healing.^4^ Upon injury, TGF‐β1 is secreted from the epithelium into the ECM in a biologically latent form called latent TGF‐β1 (L‐TGF‐β1) and is biologically inactive.^5^ It is activated by various activators such as integrins and proteases.^6^ Additionally, TGF‐β1 is secreted into tears from the conjunctiva and lacrimal gland. Upon injury to the cornea that extends deeper than the epithelium, a layer of randomly arranged collagen fibres (called Bowman's layer), which normally limits the passage of TGF‐β1 further down to the stroma, is destroyed. This event results in TGF‐β1 penetration into the stroma and initiation of the wound healing process.^7^ Transforming growth factor‐β1 induces excessive production of ECM components such as collagens I and III^8^, ^9^, ^10^ and fibronectin (FN)^11^, ^12^ by activated fibroblasts. Moreover, TGF‐β1 promotes differentiation of fibroblasts into myofibroblasts.^13^ In contrast to keratocytes, which are quiescent cells of the corneal stroma, and which main function is to sustain components of the ECM,^14^ myofibroblasts produce strong contractile force in order to close the injured tissue,^15^ and they express markers such as alpha‐smooth muscle actin (α‐SMA), vimentin and desmin.^16^ Additionally, myofibroblasts are opaque due to decreased expression of corneal crystallins, such as aldehyde dehydrogenase class 1 (ALDH1),^17^ and produce disorganized ECM, which makes them responsible for corneal haze 17, 18, 19 and lowered mechanical properties of the cornea.^20^ Contractile activity and expression of α‐SMA decrease when the injured tissue is properly healed, and myofibroblasts undergo apoptosis.^21^ However, as mentioned above, in pathological process of healing, the myofibroblasts do not cease their activity, which in turn leads to formation of fibrotic tissue.^16^

Acetylcholine (ACh) has been regarded as a classical neurotransmitter, released by cholinergic neurons and acting through activation of nicotinic and muscarinic receptors (n‐ and mAChRs).^22^ However, for the past decades, increasing evidence has shown that ACh is synthesized by a majority of human cells and that it modulates various cellular processes.^23^, ^24^, ^25^, ^26^ For example, stimulation of ACh receptors has been found to have an anti‐inflammatory effect,^27^, ^28^, ^29^ to induce proliferation markers in keratinocytes^30^ and to stimulate skin wound healing.^30^ In the cornea, the corneal epithelium has one of the highest concentrations of ACh in the body,^31^, ^32^ and it has been suggested that ACh might accelerate corneal re‐epithelialization^33^, ^34^ and play a role in migration of corneal epithelial cells.^35^ On the contrary, the concentration of ACh in the corneal stroma is very low, but our previous studies have shown that the resident keratocytes are able to produce and secrete ACh in in vitro settings.^36^ We have also demonstrated that ACh induces proliferation of keratocytes^37^ and that it decreases keratocyte apoptosis in a Fas‐ligand apoptosis model.^38^ Research on the role of ACh in fibrosis has been mostly conducted on its role in airway diseases, for which it has been shown that stimulation of mAChRs may be involved in remodelling processes in chronic airway diseases^39^ and that α7 nAChR is a key regulator of lung fibrogenesis.^40^ It has also been shown that ACh induced collagen expression and proliferation of myofibroblasts in hepatic stellate cells.^41^

At present, however, it is not known what effects, if any, ACh has on the differentiation of corneal fibroblast into myofibroblasts, nor on the development of fibrosis in the cornea. In this project, we studied the role of ACh in the transition of corneal fibroblasts into myofibroblasts, and therefore its role in the onset of fibrosis, in an in vitro human corneal fibrosis model.

## 2 MATERIALS AND METHODS

### 2.1 Human corneas

Healthy human corneas, which were obtained from deceased individuals who had chosen, when alive, to donate their corneas for transplantation or research purposes according to the Swedish law, were stored in the Tissue Establishment, Eye Bank Umeå, at the University Hospital of Umeå, Sweden, and delivered to the research laboratory if they were not used for transplantation. The project was vetted by the Regional Ethical Review Board in Umeå, which determined it to be exempt from the requirement for approval (2010‐373‐31M). The study adhered to the tenets of the Declaration of Helsinki.

### 2.2 Isolation and culture of primary keratocytes

Primary keratocytes were isolated from 14 donors. The isolation and culture of primary keratocytes have been described previously.^36^ Shortly, in order to remove remaining epithelial and endothelial cells, the corneas were scraped with a scalpel. Next, the central part of the cornea was cut out and minced with a scalpel. Corneal pieces were then digested with 2mg/mL collagenase (Sigma‐Aldrich, # C0130) diluted in DMEM/F‐12 + GlutaMAX™ medium (Thermo Fisher Scientific, Waltham, MA, USA, # 31330‐095) containing 2% foetal bovine serum (FBS; Thermo Fisher Scientific, # 10082‐147) and 1% penicillin‐streptomycin (Thermo Fisher Scientific, # 15140‐122) (DMEM/F‐12 2% FBS) overnight at 37˚C. Samples were centrifuged at 1500 rpm for 5 min. In order to keep the right phenotype and function of primary keratocytes (quiescent keratocytes), the pellet was resuspended in DMEM/F‐12 2% FBS, as described in our previous publication,^36^ in which we demonstrated, through immunocytochemistry and Western blot analyses, that keratocytes grown in DMEM/F‐12 2% FBS express specific keratocyte markers, such as CD34, keratocan, lumican and ALDH. In order to differentiate primary keratocytes into fibroblasts, for further use in the in vitro fibrosis model, the pellet was resuspended in DMEM/F12 10% FBS. Cells were cultured at 37˚C with 5% CO_2_ until they reached confluency, with medium being changed every second or third day. 0.05% trypsin‐EDTA (Thermo Fisher, # 15400‐054) was used to detach the cells. Cells were split into a 1:2 ratio for propagation. Central keratocytes and fibroblasts in passage 4 were used throughout this study. DMEM/F‐12 2% FBS or DMEM/F‐12 10% FBS was used to propagate the cell cultures. DMEM/F‐12 0.1% FBS was used to assess the role of ACh on production of ECM by quiescent keratocytes. DMEM/F‐12 10% FBS was used for the in vitro corneal fibrosis model. The corneas were assessed individually; that is, keratocytes isolated from different corneas were not pulled together.

### 2.3 Cell viability assay

The effect of ACh on the viability of corneal fibroblasts during the onset of fibrosis was measured using MTS assay (CellTiter 96® Aqueous One Solution Cell Proliferation Assay; Promega #G3580) according to the manufacturer's instructions. Briefly, corneal fibroblasts were seeded at a density of 2 × 10^3^/well in a 96‐well plate and incubated overnight. Next day, fibrosis was induced, and desired wells were concurrently treated with 10^−7^M or 10^−8^M ACh. Data were collected at times 0, 2 days and 4 days after treatment.

### 2.4 BrdU incorporation ELISA

The effect of ACh on corneal fibroblast proliferation was performed by measurement of BrdU incorporation in newly synthesized cellular DNA according to the manufacturer's instructions (R&D, #11647229001). Briefly, corneal fibroblasts were seeded at a density of 2 × 10^3^/well in a 96‐well plate and incubated overnight. Next day, fibrosis was induced, and desired wells were concurrently treated with treated with 10^−7^M or 10^−8^M ACh. Data were collected at times 0, 2 days and 4 days after treatment.

### 2.5 In vitro human corneal fibrosis model

Corneal fibroblasts were plated on plastic culture dishes at desired densities (depending on the experimental method used; cf. each method in Materials and Methods section for the exact cell density) in DMEM/F‐12 10% FBS medium and incubated overnight. Next, cells were stimulated by a stable vitamin C derivative L‐ascorbic acid 2‐phosphate sesquimagnesium salt hydrate (VitC; Sigma‐Aldrich # A8960) at a concentration of 0.5mM and 0.25ng/mL recombinant human TGF‐β1 (R&D Systems, # 240‐B) for up to 4 days. Fresh medium supplied with VitC and TGF‐β1 was supplied every 2 days. The concentration of both VitC and TGF‐β1 was based on the fibrosis model developed by Karamichos et al^42^ and was also described in our paper on the role of substance P in corneal fibrosis.^43^ The expression and secretion of ECM components and fibrotic markers collagen I, collagen III, collagen V, lumican, FN and α‐smooth muscle actin were assessed by Western blot, RT‐qPCR, ELISA and flow cytometry 2 and 4 days after stimulation. For RT‐qPCR analysis, expression of genes at 2 and 4 days was compared to time point 0. This control was set to fold 1 in the analysis, and expression of genes is represented as a fold change compared to time 0. Gel contraction assay was used to assess the contractile abilities of newly formed myofibroblasts. Unstimulated fibroblasts in DMEM/F‐12 10% FBS served as a control.

### 2.6 Persistent fibrosis

Corneal fibroblasts were plated on plastic culture dishes at desired densities (depending on the experimental method used; cf. each method in Materials and Methods section for the exact cell density) in DMEM/F‐12 10% FBS medium and incubated overnight. Next, cells were stimulated by a stable vitamin C derivative L‐ascorbic acid 2‐phosphate sesquimagnesium salt hydrate (VitC; Sigma‐Aldrich # A8960) at a concentration of 0.5 mM, and 0.25 ng/mL recombinant human TGF‐β1 (R&D Systems, # 240‐B) for 2 days. At day 2, cells were treated with either 10^−7^M or 10^−8^M ACh in DMEM/F‐12 10% FBS + TGF‐β1 + VitC. Treatment lasted for 8 days, with fresh medium and treatment supplied every 2 days. Samples were collected at 2, 4, 6 and 8 days after treatment with ACh. RT‐qPCR for ACTA2, FN, COL1A1, COL3A1 and COL5A1, and ELISA for fibronectin and pro‐collagen I were performed. For RT‐qPCR analysis, the ACh‐treated cells were compared with untreated cells at each time point. The ELISA analysis is a cumulative amount of the proteins secreted over the period of 8 days.

### 2.7 RT‐qPCR

Primary keratocytes were seeded into 6‐well plates at a density of 0.25 × 10^6^ cells per well in DMEM/F‐12 0.1% FBS one day before treatment. Cells were treated with ACh at concentrations of either 10^−7^M or 10^−8^M for up to 8 days with fresh medium replacement and treatment repeated every 2 days. Gene expression of the experimental samples was compared to an untreated time 0 control for the analysis. Corneal fibroblasts were seeded into 6‐well plates at a density of 0.25 × 10^6^ cells per well in DMEM/F12 10% FBS one day before the treatment. Fibrosis was induced in the cells as described in the previous section. At the same time, desired wells were also treated with ACh at concentrations of either 10^‐7^M or 10^‐8^M for up to 4 days with fresh medium containing VitC and TGF‐β1 replaced and treated repeated every 2 days. Gene expression of the experimental samples was compared with fibroblasts in DMEM/F12 10% FBS at time 0. Cells were lysed at 2, 4 and 8 days (keratocytes only), and mRNA was extracted using the RNA extraction kit (Qiagen # 74106) according to the manufacturer's instructions. Next, 1 000 ng of RNA was reverse‐transcribed into cDNA using the high‐capacity cDNA reverse transcription kit (Thermo Fisher, # 4368813). Collagen I (COL1A1), collagen III (COL3A1), collagen V (COL5A1), lumican (LUM), fibronectin (FN) and α‐smooth muscle actin (ACTA2) probes were used in order to determine gene expression (Thermo Fisher). Samples were run in duplicates in ViiA™ 7 Real‐Time PCR System (Thermo Fisher). 18S and β‐actin probes served as endogenous controls (Thermo Fisher; # 4333760F and # 4352935E, respectively). For analysis, each time point was compared to time 0 (set to fold 1). Analysis was performed with ViiA™ 7 Software (Thermo Fisher).

### 2.8 Western blot

Primary keratocytes were seeded into 6‐well plates at a density of 0.25 × 10^6^ cells per well in DMEM/F‐12 0.1% FBS one day before treatment. Cells were treated with ACh at concentrations of either 10^−7^M or 10^−8^M for up to 8 days with fresh medium replacement and treatment repeated every 2 days. Corneal fibroblasts were seeded into 6‐well plates at a density of 0.25 x 10^6^ cells per well in DMEM/F12 10% FBS one day before the treatment. Fibrosis was induced in the cells as described in the previous section. At the same time, desired wells were also treated with ACh at concentrations of either 10^−7^M or 10^−8^M for up to 4 days with fresh medium containing VitC and TGF‐β1 replaced and treated repeated every 2 days. Cells were freeze‐thawed three times and further lysed in RIPA (radioimmunoprecipitation) lysis buffer (Thermo Fisher, # 89901) supplemented with protease and phosphatase inhibitor cocktail (Thermo Fisher, # 78446) at 2, 4 and 8 days (keratocytes only), and total protein concentration was determined with Bradford assay (Bio‐Rad # 5000006). Samples containing 30 µg of protein were separated on SDS‐polyacrylamide gels (Bio‐Rad) and transferred to PVDF membranes (GE Healthcare # GEHERPN303F). Next, membranes were blocked with 5% (w/v) bovine serum albumin (BSA; Sigma‐Aldrich, # A9647) in TRIS‐buffered saline (TBS) containing 0.1% Tween‐20 (TBS‐T; VWR, # 28829.183) for one hour at room temperature and incubated overnight at 4˚C with rabbit polyclonal anti–alpha‐smooth muscle actin (α‐SMA) antibody from Abcam (# ab5694) or rabbit anti‐β‐actin antibody from Cell Signaling (# 4967). Next, membranes were washed in TBS‐T and incubated with anti‐rabbit IgG, HRP‐linked secondary goat anti‐rabbit antibody from Cell Signaling (# 7074) for one hour at room temperature. Images were taken by Odyssey® Fc imaging system (LI‐COR).

### 2.9 ELISA

1 × 10^4^ keratocytes were seeded into 96‐well plates in DMEM/F‐12 0.1% medium one day prior to the treatment with either 10^−7^M or 10^−8^M ACh. To study whether ACh acts through activation of muscarinic receptors, appropriate wells were pre‐treated with atropine (Sigma‐Aldrich, # A0132), the muscarinic receptor inhibitor, at a concentration of 10^−5^M for 20 min. Medium was replaced, and treatment was repeated every 2 days. Supernatants and cell lysates were collected at 4 h, 1 day, 2 days, 4 days and 8 days after treatment. 0.3 × 10^4^ corneal fibroblasts were seeded into 96‐well plates in DMEM/F‐12 10% medium one day before the treatment. Fibrosis was induced in the cells as described earlier. Appropriate wells were pre‐treated with atropine at a concentration of 10^−5^M for 20 min, and cells were treated, concurrently, with ACh at concentrations of either 10^−7^M or 10^−8^M. The treatment was repeated every 2 days. Supernatants and cell lysates were collected at 4h, 1 day, 2 days and 4 days after treatment. Secretion and intracellular production of pro‐collagen I was assessed with Human Pro‐Collagen I alpha 1 DuoSet ELISA kit (R&D Systems, #DY6220), collagen III with Human Collagen, type III, alpha 1 (COL3A1) ELISA kit (Cusabio # CSB‐E13446h), collagen V with Human Collagen, type V, alpha 1 (COL5A1) ELISA kit (Cusabio, # CSB‐E13447h), FN with Human Fibronectin DuoSet ELISA kit (R&D Systems, # DY1918), and lumican with Human Lumican DuoSet ELISA kit (R&D Systems, # DY2846) according to the manufacturer's protocol.

### 2.10 Contraction assay

500 µL (per well of a 24‐well culture plate) of bovine collagen solution, type I PureCol® in DMEM/F‐12 medium, 5 mg/mL (0.5%) (Advanced BioMatrix # 5074‐35ML) liquid gel was mixed together with 0.25 × 10^6^ corneal fibroblasts, FBS (10%), 0.5 mM VitC and 0.25 ng/mL recombinant human TGF‐β1. The experimental group gels contained ACh at concentration of either 10^−7^M or 10^−8^M, atropine at concentration of 10^−5^M or 10^−5^M atropine with either 10^−7^M or 10^−8^M ACh. Gels containing cells and 10% FBS served as controls. Gels were left to polymerize for one hour at 37˚C. Afterwards, DMEM/F‐12 10% FBS containing 0.5 mM VitC and 0.25 ng/mL recombinant human TGF‐β1 was added to the experimental groups, on top of the gels. Acetylcholine at concentration of either 10^−7^M or 10^−8^M, atropine at concentration of 10^−5^M or 10^−5^M atropine with either 10^−7^M or 10^−8^M ACh was added to the medium in corresponding wells. DMEM/F‐12 containing 10%FBS only was added to the control wells. Next, gels were detached from the wells and photographed at 0 h, 4 h, 1 day, 2 days, 3 days and 4 days. The areas of the contracted gels were measured using Photoshop (Adobe Systems). Each treatment was performed in triplicates. Mean area was calculated for each experimental group at each time point. The results are shown as a per cent of contraction: % contraction = (initial area – final area)/(initial area) × 100%.

### 2.11 Flow cytometry

Flow cytometry was used to assess expression of alpha‐smooth muscle actin in the fibrosis model. 0.25 × 10^6^ corneal fibroblasts were seeded into 6‐well plates in triplicates in DMEM/F‐12 10% FBS medium one day before treatment. Fibrosis was induced as described in the earlier section. Cells were harvested with 0.05% trypsin‐EDTA 2 days and 4 days after treatment, and washed two times with PBS. 1 × 106 cells/100 µL was aliquoted into FACS tubes (VWR #734‐0442). 0.5 mL of cold Flow Cytometry Fixation Buffer (R&D, #FC004) was added to each tube. Tubes were vortexed and incubated for 10 min at room temperature. Next, cells were washed two times with PBS and the pellet was resuspended in 200 µL of Flow Cytometry Permeabilization/Wash Buffer I (R&D, #FC005). 10 µL of human alpha‐smooth muscle actin PE‐conjugated antibody (R&D Systems # IC1420P) was added. Cells were vortexed and incubated for 30 min at room temperature in the dark. Afterwards, cells were washed two times with Flow Cytometry Permeabilization/Wash Buffer I and pellet was resuspended in 400 µL of PBS for flow cytometric analysis. Cells were analysed with BD LSR II flow cytometer (Becton Dickinson) and FlowJo (FlowJo LLC).

### 2.12 Scratch assay

0.4 × 10^6^ keratocytes or 0.2 × 10^6^ corneal fibroblasts were seeded into six‐well plates in DMEM/F‐12 0.1% FBS (keratocytes) or DMEM/F‐12 10% FBS (corneal fibroblasts) and allowed to adhere overnight. Next morning, bottom of all wells was scratched with a 200‐μL pipette tip, creating a wound field. Cells were washed with PBS. Keratocytes were treated with either 10^−7^M or 10^−8^M ACh in DMEM/F‐12 0.1% FBS. Fresh medium replacement and treatment were performed every two days. Fibrosis was induced in corneal fibroblasts as described in the previous sections. At the same time, desired wells were also treated with ACh at concentrations of either 10^−7^M or 10^−8^M. Images of the wounds were taken at same spots, at times 0 h, 1 day, 2 days and 4 days for keratocytes and at times 0h, 1 day and 2 days for corneal fibroblasts using Motic AE31 trinocular inverted microscope (Richmond, BC, Canada). Migration was assessed with MRI Wound Healing tool of ImageJ software (NIH). The areas of the wound were calculated and presented as % of closed wound.

### 2.13 Statistical analysis

All experiments were performed in triplicates. Data are presented as mean ± SD. Statistical analysis was performed with one‐way ANOVA, with Tukey's post hoc test, or unpaired t test. Differences were considered statistically significant at a *P*‐value of < .05. All experiments were performed at least three times, meaning that at least three separate experiments were performed with cells isolated from different patients (biological replicates).

## 3 RESULTS

### 3.1 Acetylcholine reduces gene expression and production of ECM components and of fibrotic markers in quiescent keratocytes

**Figure 1:**
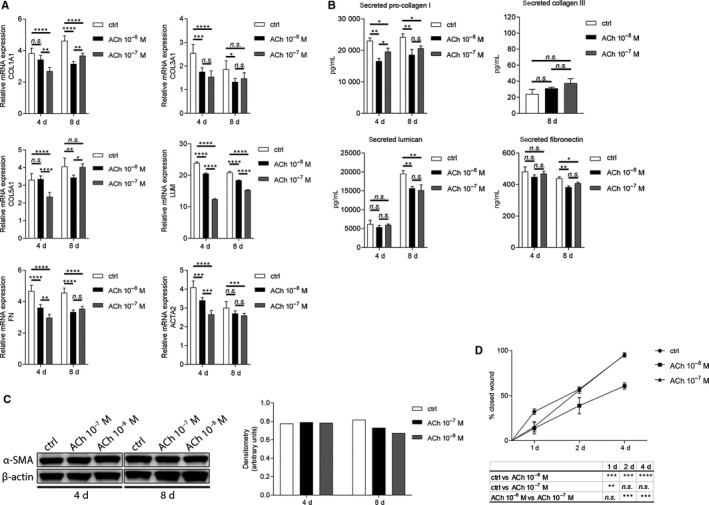
ACh decreases gene expression and production of extracellular matrix components and fibrotic markers in quiescent keratocytes. (A) qRT‐PCR analysis in human primary keratocytes showing RNA expression levels of COL1A1, COL3A1, COL5A1, LUM, FN and ACTA2 after ACh stimulation. Each time point was compared to time 0 (set to fold 1). (B) Secretion of pro‐collagen I, collagen III, lumican and fibronectin after ACh treatment was assessed by ELISA. (C) Western blot analysis showing the effect of ACh on α‐SMA (42 kDa) expression in human primary keratocytes. β‐Actin (45 kDa) served as loading control. (D) Scratch assay showing % of closed wound after ACh stimulation at specific time points. Densitometry analysis and scratch assay evaluation were performed with ImageJ software. Values are means ± SD. n.s. (not significant); *P < .05; **P < .01; ***P < .001; ****P < .0001

First, we wanted to establish whether ACh has an effect on ECM components and fibrotic markers in quiescent keratocytes. The effect of ACh on production of collagen I, collagen III, collagen V, lumican, FN and α‐SMA by quiescent keratocytes was assessed by qRT‐PCR, ELISA and Western blot. The results showed that 10^−8^M ACh decreased expression of collagen I gene (COL1A1) 8 days after treatment, whereas 10^−7^M ACh decreased COL1A1 expression both 4 days and 8 days after treatment. Collagen III (COL3A1) gene expression was decreased by both 10^−8^M and 10^−7^M ACh at day 4. Additionally, 10^−8^M ACh decreased COL3A1 expression after 8 days. Collagen V (COL5A1) gene expression decreased after both 10^−8^M and 10^−7^M ACh treatments at day 8. Moreover, 10^−7^M ACh decreased COL5A1 expression 4 days after treatment. Lumican (LUM) gene expression decreased after both 10^−8^M and 10^−7^M ACh treatments at 4 and 8 days, with 10^−7^M ACh having a more pronounced effect. Gene expression of two fibrotic markers, fibronectin (FN) and α‐SMA (ACTA2), decreased after both 10^−8^M and 10^−7^M ACh treatments at days 4 and 8, except for 10^−8^M ACh, which did not have a significant effect on day 8 (Figure 1A). Treatment of keratocytes with both 10^−8^M and 10^−7^M ACh reduced secretion of pro‐collagen I at days 4 and 8. Secreted collagen III was not detected at day 4, and its secretion was not affected by ACh treatment at day 8. No secreted collagen V could be measured at any time point. Both 10^−8^M and 10^−7^M ACh reduced secretion of lumican and FN 8 days after treatment (Figure 1B). The same effect of ACh was observed on α‐SMA protein expression; that is, both 10^−8^M and 10^−7^M ACh reduced its expression 8 days after treatment (Figure 1C). We performed a scratch assay in order to assess whether ACh has an effect on keratocyte migration. The results showed that 10^−8^M ACh decreased keratocyte migration by 40%. However, 10^−7^M ACh had no effect (Figure 1D). Additionally, intracellular levels of pro‐collagen I, collagen III, collagen V, lumican and FN were assessed. Except for 10^−8^M ACh, which decreased pro‐collagen I levels at day 8, and both 10^−8^M and 10^−7^M ACh, which reduced intracellular FN at day 4, no significant differences were found after ACh treatments (Figure S1).

### 3.2 In vitro human corneal fibrosis model

**Figure 2:**
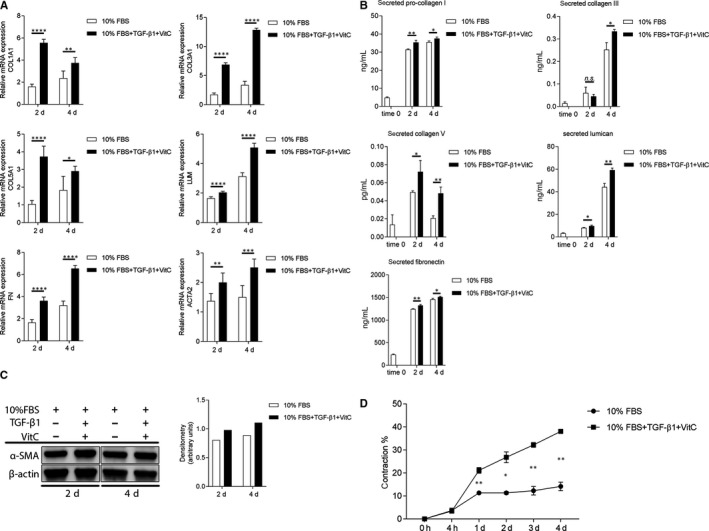
In vitro human corneal fibrosis model. (A) qRT‐PCR analysis showing RNA expression levels of COL1A1, COL3A1, COL5A1, LUM, FN and ACTA2 after induction of fibrosis in human corneal fibroblasts. Each time point was compared to time 0 (set to fold 1). (B) Secretion of pro‐collagen I, collagen III, collagen V, lumican and fibronectin after induction of fibrosis in human corneal fibroblasts was assessed by ELISA. (C) Western blot analysis of α‐SMA (42 kDa) expression after induction of fibrosis in human corneal fibroblasts. β‐Actin (45 kDa) served as loading control. Densitometry analysis was performed with ImageJ software. (D) Collagen gel contraction assay was used to determine the contractile abilities of the cells after induction of fibrosis in human corneal fibroblasts. Values are means ± SD. n.s. (not significant); *P < .05; **P < .01; ***P < .001; ****P < .0001

In order to study the effect of ACh in the onset of fibrosis and in the transition of corneal fibroblasts to myofibroblasts, we adapted the in vitro human corneal fibrosis model from Karamichos et al. As described in Materials and Methods, we treated corneal fibroblasts with 0.5 mM VitC and 0.25 ng/mL recombinant human TGF‐β1 in order to induce the fibrosis process. Untreated corneal fibroblasts served as control. We assessed various extracellular matrix and fibrotic markers in order to confirm the model. First, we determined gene expression of COL1A1, COL3A1, COL5A1, LUM, FN and ACTA2. Our results showed that expression of all the genes tested was increased after treatment with VitC and TGF‐β1 at days 2 and 4 (Figure 2A). Next, we checked for secretion of pro‐collagen I, collagen III, collagen V, lumican and FN. Again, induction of fibrosis with VitC and TGF‐β1 resulted in increased secretion of all markers mentioned at days 2 and 4, except for collagen III secretion at day 2, for which we found no difference between treated and untreated cells (Figure 2B). Expression of α‐SMA was assessed by Western blot and flow cytometry and showed that cells treated with VitC and TGF‐β1 expressed more α‐SMA protein than untreated cells both at day 2 and day 4 (Figure 2C and Figure S2F, respectively). Intracellular levels of pro‐collagen I, collagen III, collagen V, lumican and FN were increased after fibrosis induction (Figure S2). Lastly, gel contraction assay was used to assess the contractile abilities of newly formed myofibroblasts. Induction of fibrosis with VitC and TGF‐β1 resulted in significantly increased contractile abilities of the cells from day 1 to day 4 (Figure 2D). We concluded that the in vitro human corneal fibrosis model is appropriate for our further studies on the ACh effect on the onset of fibrosis.

### 3.3 Acetylcholine reduces gene expression and production of ECM components during the process of fibrosis

**Figure 3:**
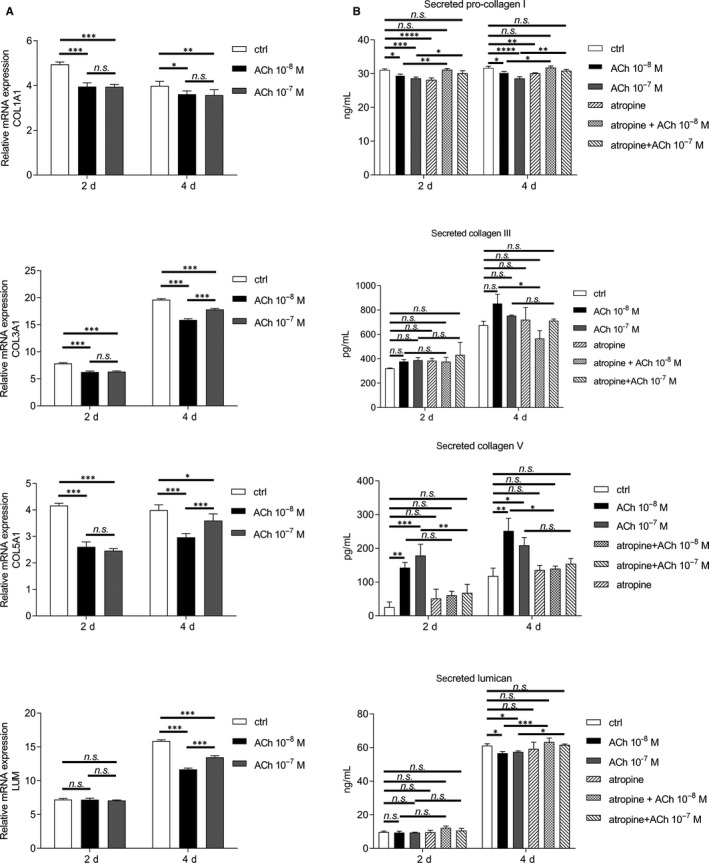
ACh decreases expression and production of extracellular matrix components during the process of fibrosis. (A) Effect of ACh on RNA expression levels of COL1A1, COL3A1, COL5A1 and LUM 2 and 4 days after induction of fibrosis in human corneal fibroblasts, as assessed by qRT‐PCR. Each time point was compared to time 0 (set to fold 1). (B) Effect of ACh and atropine on secretion of pro‐collagen I, collagen III, collagen V and lumican after induction of fibrosis in human corneal fibroblasts was determined by ELISA. Values are means ± SD. n.s. (not significant); *P < .05; **P < .01; ***P < .001; ****P < .0001

First, in order to assure that the concentrations of ACh used in the fibrosis study are not toxic and that the possible effect of ACh on the onset of fibrosis is not caused by cell death or lack of proliferation of corneal fibroblasts, we performed cell viability assays and BrdU incorporation ELISA. In accordance with our previous findings on ACh enhancing proliferation in primary keratocytes,^37^ we also observed similar effect on corneal fibroblasts during the onset of fibrosis. Both cell viability and proliferation were enhanced in ACh‐treated cells (Figure S3). Next, we wanted to assess whether ACh affects transition of fibroblasts into myofibroblasts during the fibrosis process. First, we determined the role of ACh in gene expression of ECM components collagen I, collagen III, collagen V and lumican by qRT‐PCR. The results showed that expression of COL1A1, COL3A1 and COL5A1 decreased after treatment with both 10^−8^M and 10^−7^M ACh at days 2 and 4 during the fibrosis process. Gene expression of LUM was unaffected by ACh at day 2, but it decreased at day 4 (Figure 3A). Next, we assessed secretion of pro‐collagen I, collagen III, collagen V and lumican by ELISA. We observed that secretion of pro‐collagen I was reduced after treatment with both 10^−8^M and 10^−7^M ACh at days 2 and 4. Additionally, cells were pre‐treated with 10^−5^M atropine to examine whether mAChR activation is necessary to carry the ACh effect. The results showed that ACh effect was no longer present when mAChRs were blocked. Secretion of collagen III was not affected by ACh treatment during the fibrosis process. Interestingly, secretion of collagen V increased after treatment with both 10^−8^M and 10^−7^M ACh at days 2 and 4. Blocking mAChRs with 10^−5^M atropine reduced secretion of collagen V. Secretion of lumican was not affected by ACh at day 2. However, 4 days after treatment with either 10^−8^M or 10^−7^M, secretion of lumican was reduced. This effect was abolished when mAChRs were blocked with 10^−5^M atropine (Figure 3B). Intracellular levels of pro‐collagen I and collagen III were not affected by ACh treatment. However, intracellular level of collagen V was reduced by 10^−8^M ACh at day 4, and lumican by both 10^−8^M and 10^−7^M ACh also at day 4 (Figure S4).

### 3.4 Acetylcholine decreases gene expression and production of fibrotic markers during the process of fibrosis

**Figure 4:**
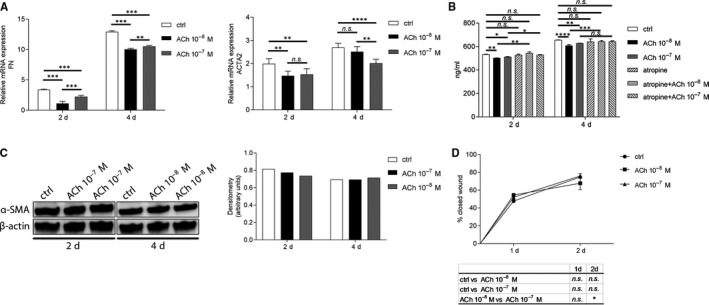
ACh decreases expression and production of fibrotic markers during the process of fibrosis. (A) Effect of ACh on RNA expression levels of FN and ACTA2 2 and 4 days after induction of fibrosis in human corneal fibroblasts, assessed by qRT‐PCR. (B) Effect of ACh and atropine on secretion of fibronectin after induction of fibrosis in human corneal fibroblasts determined by ELISA. (C) Effect of ACh on α‐SMA (42 kDa) expression after fibrosis induction in human corneal fibroblasts was analysed by Western blot. β‐Actin (45 kDa) served as loading control. (D) Scratch assay showing % of closed wound after ACh stimulation at specific time points. Densitometry analysis and scratch assay evaluation were performed with ImageJ software. Values are means ± SD. n.s. (not significant); *P < .05; **P < .01; ***P < .001; ****P < .0001

Next step in assessing the role of ACh in transitioning of fibroblasts into myofibroblasts during the fibrosis process was to determine whether ACh affects gene expression of two fibrotic markers: FN and ACTA2. The results showed that gene expression of FN decreased after both 10^−8^M and 10^−7^M ACh treatment at days 2 and 4. 10^−8^M ACh decreased FN expression significantly more than 10^−7^M ACh. ACTA2 expression decreased after 10^−7^M ACh treatment at day 2 and day 4. 10^−8^M ACh decreased ACTA2 expression only 2 days after treatment (Figure 4A). Secretion of FN was assessed by ELISA and showed that both 10^−8^M and 10^−7^M ACh reduced FN secretion at days 2 and 4. Blocking mAChRs with 10^−5^M atropine abolished the effect of ACh (Figure 4B). Additionally, intracellular level of FN was decreased by 10^−7^M ACh at day 2 (Figure S4E). α‐SMA protein expression was assessed by Western blot. The results showed that both 10^−8^M and 10^−7^M ACh reduced α‐SMA expression at day 2. Acetylcholine had no effect 4 days after ACh treatment (Figure 4C). Additionally, scratch assay was performed to assess the effect of ACh on fibroblast migration during the onset of fibrosis. The results showed that neither 10^−8^M ACh nor 10^−7^M ACh had an effect on fibroblast migration (Figure 4D).

### 3.5 Acetylcholine inhibits corneal fibroblasts from developing contractile abilities

**Figure 5:**
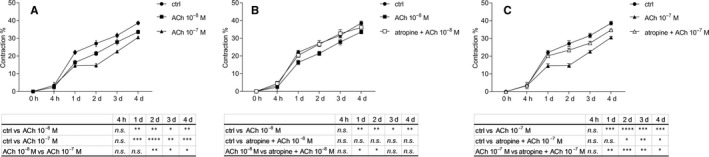
ACh inhibits corneal fibroblasts from developing contractile abilities. Collagen gel contraction assay was used to determine the effect of ACh (A) and atropine (B, C) on contractile abilities of the cells after induction of fibrosis in human corneal fibroblasts. Values are means ± SD. n.s. (not significant); *P < .05; **P < .01; ***P < .001; ****P < .0001

As our results suggest that ACh decreases transformation of corneal fibroblasts into myofibroblasts during the fibrosis process, we opted for determining whether ACh would also decrease contractile abilities of the transitioning cells. To achieve that, we used a collagen I contraction assay. We tested two concentrations of ACh: 10^−8^M and 10^−7^M. Both concentrations of ACh significantly decreased gel contraction from day 1 to day 4. Moreover, 10^−7^M ACh decreased gel contraction significantly more than 10^−8^M ACh from day 2 (Figure 5A). Next, we blocked mAChRs with 10^−5^M atropine and treated cells with 10^−8^M ACh, and could observe that gel contraction increased as compared to cells treated with ACh only, and it achieved the level of the control (Figure 5B). Likewise, blocking mAChRs and treating cells with 10^−7^M ACh resulted in significantly increased gel contraction when compared to 10^−7^M ACh‐only treatment (Figure 5C).

### 3.6 Acetylcholine decreases gene expression and production of fibrotic markers in persistent fibrosis

**Figure 6:**
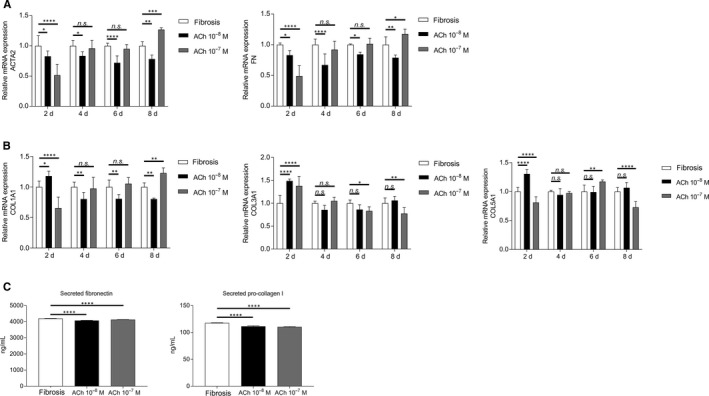
ACh decreases gene expression and production of fibrotic markers in persistent fibrosis. (A) Effect of ACh on RNA expression levels of ACTA2 and FN in persistent fibrosis setting 2 days, 4 days, 6 days, and 8 days after ACh treatment, assessed by qRT‐PCR. (B) Effect of ACh on RNA expression levels of COLA1A, COL3A1 and COL5A1 in persistent fibrosis setting 2, 4, 6 and 8 days after ACh treatment, assessed by qRT‐PCR. (C) Cumulative secretion of fibronectin and pro‐collagen I after ACh treatment of persistent fibrosis for a period of 8 days, determined by ELISA. Values are means ± SD. n.s. (not significant); *P < .05; **P < .01; ***P < .001; ****P < .0001

We were interested in whether ACh exhibits the same anti‐fibrotic effect in a setting where the fibrosis has already occurred. Therefore, we induced fibrosis in corneal fibroblasts for 2 days. Afterwards, cells were treated with 10^−8^M or 10^−7^M ACh for 8 days. The results showed that 10^−8^M ACh significantly decreased gene expression of both ACTA2 and FN for the period of 8 days. However, 10^−7^M ACh decreased gene expression only 2 days after treatment, whilst at day 8, the gene expression was increased (Figure 6A). Acetylcholine in the concentration 10^−8^M decreased gene expression of COL1A1; however, it did not have an effect on gene expression of COL3A1 and COL5A1. 10^−7^M ACh decreased COL1A1 gene expression only 2 days after treatment, whilst at day 8, the gene expression was increased. We observed an opposite effect of 10^−7^M ACh on gene expression of COL3A1 and COL5A1; that is, it increased the gene expression only 2 days after treatment, whilst at day 8, the gene expression was decreased (Figure 6B). Additionally, cells treated with ACh secreted significantly less pro‐collagen I and fibronectin over the period of 8 days (Figure 6C). Expression of α‐SMA was not significantly altered by the ACh treatment (data not shown).

## 4 DISCUSSION

This study shows that ACh has a negative effect on ECM component production both in quiescent keratocytes and during the onset of fibrosis in an in vitro human corneal fibrosis model. Moreover, ACh down‐regulates expression of fibrotic markers and it diminishes the transition of corneal fibroblasts into myofibroblasts.

First, we wanted to study whether ACh has an effect on production of ECM components and fibrotic marker expression in quiescent keratocytes. Based on our previous studies on ACh and keratocytes,^37^, ^38^ we tested two concentrations of ACh: 10^−7^M and 10^−8^M. Our results showed that ACh decreased gene expression of all markers tested, with 10^−7^M of ACh having bigger effect at earlier time point. Moreover, ACh also decreased secretion of pro‐collagen I. We chose to determine the secretion of pro‐collagen I instead of collagen I as it has been reported that keratocytes in cell culture are not able to process all pro‐collagen I to mature collagen I, and as a result, pro‐collagen I accumulates in the cell medium with only a small portion being processed to mature collagen I.^44^ Moreover, ACh had no effect on collagen III secretion and we could not detect secreted collagen V. Interestingly, we have shown that ACh stimulates keratocytes to proliferate^37^; therefore, we hypothesized that ACh would possibly increase expression of ECM and fibrotic markers. Surprisingly, our results showed the contrary, and the reason for that should be studied.

The role of ACh in fibrosis has been investigated in airway diseases and in liver fibrosis, for which it has been shown that ACh is involved in ECM remodelling and proliferation of myofibroblasts,^39^, ^40^, ^41^ but no data exist on its possible role in corneal fibrosis. We have adapted an in vitro human corneal fibrosis model from Karamichos et al^42^ in order to study the role of ACh in the onset of fibrosis and its effect on the transition of fibroblasts to myofibroblasts. In this model, vitamin C and TGF‐β1 are used to induce fibrotic process in corneal fibroblasts. Vitamin C has been shown to induce synthesis and secretion of ECM components,^45^ especially collagen I, without altering non‐collagen protein synthesis such as fibronectin.^46^ Transforming growth factor‐β1 stimulates overproduction and deposition of ECM.^8^, ^9^, ^10^ Using this model, we were able to induce overproduction of ECM components (collagen I, collagen III, collagen V and lumican) and expression of the fibrotic markers FN and α‐SMA in newly formed myofibroblasts, which presence was confirmed by gel contraction assay.

Again, our results showed that ACh had an inhibitory effect on the formation of fibrosis and myofibroblasts in our model. It down‐regulated gene expression of collagen I, collagen III, collagen V and lumican. Secretion of pro‐collagen I and lumican was decreased; however, secretion of collagen III was unaffected by ACh. Interestingly, secretion of collagen V was enhanced by ACh. However, the results showed that the intracellular content of collagen V was decreased by ACh. Perhaps, the ACh‐treated cells were able to secrete collagen V more rapidly, but the total content of collagen V (intracellular + secreted) was unaffected by ACh treatment, as was the case for collagen III, for which ACh had no effect on either its secretion or its intracellular levels. Moreover, ACh decreased gene expression and secretion of FN, which might explain the lower levels of secreted collagen I, as FN is responsible for its deposition during fibrosis.^47^ We have found no apparent difference in action between the two concentrations of ACh used. Our results also suggest that the anti‐fibrotic effect of ACh is mediated by activation of mAChRs. In our previous study, we found that ACh treatment in keratocytes activates mAChRs rather than nAChRs,^37^ in order to enhance keratocyte proliferation. Perhaps, the activation of mAChRs, rather than nAChRs, in keratocytes and corneal myofibroblasts leads to an anti‐fibrotic response, rather than a pro‐fibrotic one, as it has been reported for airways that activation of α7 nAChR is involved in the progression of lung fibrosis.^39^, ^40^ Studies have shown contradicting results regarding the role of the mAChR antagonist atropine in fibrosis. Atropine has been found to have an anti‐fibrotic effect in hepatocytes isolated from rat fibrotic liver^48^; however, others have shown that atropine has pro‐fibrotic properties in rat cardiac fibroblasts.^49^ Our results suggest that atropine alone does not affect the fibrotic process in corneal fibroblasts. However, it is possible that it affects collagen secretion.

Finally, we showed that ACh hinders transition of corneal fibroblasts into myofibroblasts, as demonstrated by down‐regulation of α‐SMA and subsequent inhibition of development of contractile activity by the fibroblasts. Again, this effect was shown to be mediated by activation of mAChRs, since the usage of atropine increased the contractile activity of the cells. The effect of ACh was dose‐dependent with higher concentration inhibiting the contraction more. Importantly, we showed that ACh (only the lower concentration) decreased keratocyte migration, which further supports our hypothesis and shows that ACh has an effect under more physiological settings, as mechanical wounds or chemical burns are one of the most common reasons for corneal injury. We think that during injury, ACh affects the keratocytes in a way that they remain quiescent or that they are activated in a slower manner. The reason for ACh not having an effect on fibroblast migration during the onset of fibrosis is perhaps because of the experimental settings, that is very high amount of FBS, which under physiological conditions would be omitted.

The small, but significant, decrease in fibrotic markers by ACh observed in this study could have potential clinical application. The physiological conditions differ greatly from the in vitro settings, and at least up to this date, it is hard to reproduce the physiological conditions. Issues such as substrate stiffness will affect the in vitro experiments, and isolation of the cells from their natural environment will, to a degree, change them too.^50^, ^51^ We expected to see a significantly bigger decrease in α‐SMA expression, as contraction of the collagen gels, in which the corneal fibroblasts are embedded, was decreased greatly by the ACh‐treated cells. Perhaps, α‐SMA removal from the stress fibres is somehow deficient, or α‐SMA is not properly degraded. One study showed that when inducing apoptosis in fibroblasts, α‐SMA was degraded by caspase‐3.^52^ We have previously shown that ACh has an anti‐apoptotic effect on keratocytes.^38^ Therefore, we could speculate that a similar mechanism inhibits α‐SMA degradation in this study. Additionally, as ACh increases proliferation of corneal fibroblasts during the onset of fibrosis, the small changes observed could be a result of that. Perhaps treating the cells with ACh after arresting the cell cycle could result in a bigger and clearer change.

Taken together, our results suggest that ACh displays anti‐fibrotic characteristics in an in vitro human corneal fibrosis model. ACh impedes overproduction of ECM components and hampers expression of fibrotic markers, and this effect is driven by activation of mAChRs. Therefore, ACh not only might play a regulating role during the initial stages of corneal fibrosis, but also might decrease an already‐existing fibrosis. Hence, perhaps it contributes to reduced scarring of the cornea. Our findings are promising, as it has been reported by Uberti et al^53^, ^54^ that kinetically energized ultra‐low doses of ACh show remarkably great wound healing properties both in vitro in human keratinocytes and in vivo in mice. Even though our results cannot be directly compared with Uberti's findings, throughout our studies on ACh in corneal wound healing, we have observed that the lower dose of ACh exerts stronger wound healing and anti‐fibrotic effects. Moreover, it seems that the kinetically energized ultra–low‐dose ACh is safe for topical applications to skin in pre‐clinical studies, which would be very promising and beneficial for treating corneal wounds. Additionally, it might be interesting to study whether application of anti‐cholinergic drugs such as tropicamide, atropine or cyclopentolate, which are commonly administered for the purpose of ocular examination in order to dilate the pupil,^55^ or application of cholinergic drugs such a pilocarpine, which is used to constrict the pupil in the treatment of angle closure glaucoma,^56^ will have a negative or positive effect, respectively, on persons with wounded corneas.

## CONFLICT OF INTEREST

The authors confirm that there are no conflicts of interest.

## AUTHOR CONTRIBUTION

MS and PD participated in research design. MS performed the experiments. MS analysed the data. MS and PD wrote the paper.

## Supporting information

Fig S1‐S4

## Data Availability

The data used in the current study are available from the corresponding author on reasonable request.
