# Supplementary material for: Acetylcholine decreases formation of myofibroblasts and excessive extracellular matrix production in an in vitro human corneal fibrosis model
Source: J Cell Mol Med. 2020 Mar 16;24(8):4850–62. doi: 10.1111/jcmm.15168 (PMC7176861; doi:10.1111/jcmm.15168)
Supplement: Supplementary file 1 — Fig S1‐S4 [file JCMM-24-4850-s001.docx]

SUPPLEMENTARY MATERIAL

SUPPLEMENTARY FIGURE 1


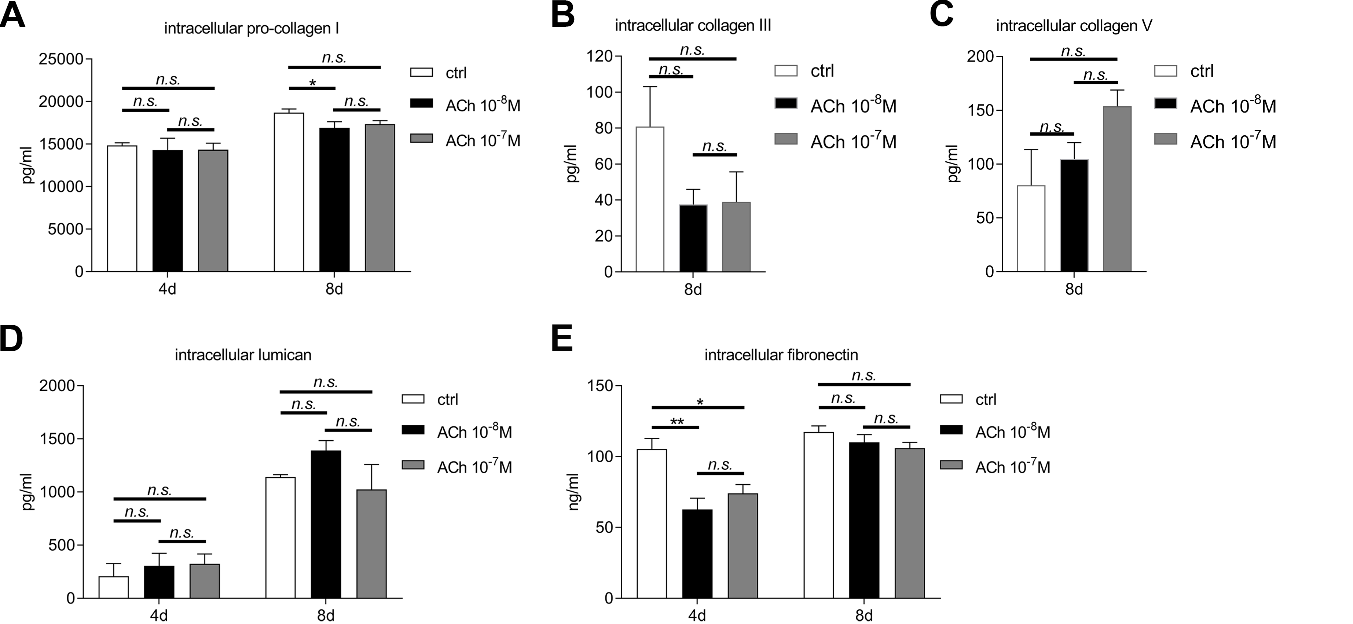


Supplementary Figure 1. **Intracellular levels of extracellular matrix components and fibrotic markers in quiescent keratocytes after ACh treatment.** The effect of ACh on intracellular levels of pro-collagen I (A), collagen III (B), collagen V (C), lumican (D), and fibronectin (E) in quiescent keratocytes were assessed by ELISA. (**A**) (**B**) (**C**) (**D**) Values are means ± SD. n.s. (not significant); *p<0.05; **p<0.01.

SUPPLEMENTARY FIGURE 2


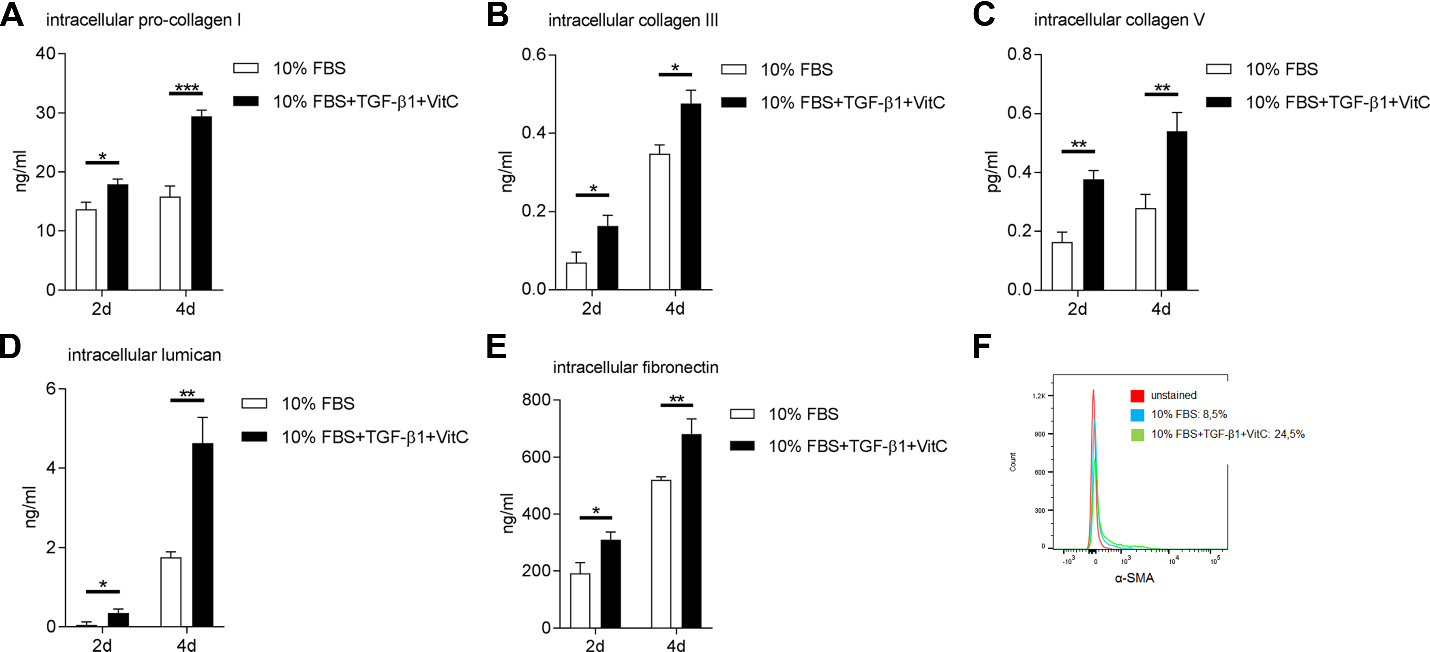


Supplementary Figure 2. **Intracellular levels of extracellular matrix components and fibrotic markers during fibrotic process.** Intracellular levels of pro-collagen I (A), collagen III (B), collagen V (C), lumican (D), and fibronectin (E) after induction of fibrosis in human corneal fibroblasts were assessed by ELISA. (F) Expression of α-SMA in human corneal fibroblasts after induction of fibrosis as determined by flow cytometry. Values are means ± SD. *p<0.05; **p<0.01; ***p<0.001.

SUPPLEMENTARY FIGURE 3





Supplementary Figure 3. **The effect of ACh on cell viability and proliferation during the onset of fibrosis.** The effect of ACh on the viability of corneal fibroblasts was assessed by MTS assay (A). The effect of ACh on the proliferation of corneal fibroblasts was assessed by BrdU incorporation ELISA (B). Values are means ± SD. n.s. (not significant); *p<0.05; **p<0.01, ***p<0.001; ****p<0.0001.

SUPPLEMENTARY FIGURE 4


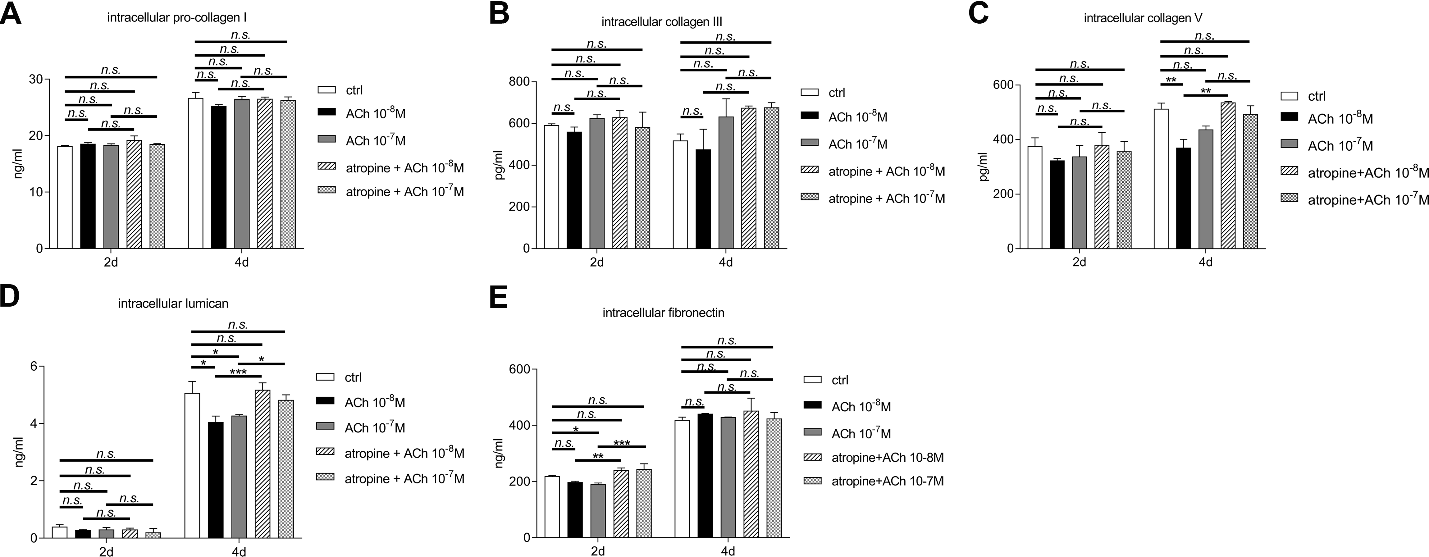


Supplementary Figure 4. **Intracellular levels of extracellular matrix components and fibrotic markers during fibrotic process after ACh treatment.** The effect of ACh and atropine on intracellular levels of pro-collagen I (A), collagen III (B), collagen V (C), lumican (D), and fibronectin (E) in human corneal fibroblasts after fibrosis induction were assessed by ELISA. Values are means ± SD. n.s. (not significant); *p<0.05; **p<0.01; ***p<0.001.
